# Biosensors Based on Nano-Gold/Zeolite-Modified Ion Selective Field-Effect Transistors for Creatinine Detection

**DOI:** 10.1186/s11671-017-1943-x

**Published:** 2017-03-02

**Authors:** Berna Ozansoy Kasap, Svitlana V. Marchenko, Oleksandr O. Soldatkin, Sergei V. Dzyadevych, Burcu Akata Kurc

**Affiliations:** 1 Department of Micro and Nanotechnology, Middle East Technical University, Ankara, 0631 Turkey; 2 Laboratory of Biomolecular Electronics, Institute of Molecular Biology and Genetics, National Academy of Sciences of Ukraine, 150 Zabolotnogo Str., 03680 Kyiv, Ukraine; 3 Institute of High Technologies, Taras Shevchenko National University of Kyiv, 64 Volodymyrska St., Kyiv, 01003 Ukraine; 4 Central Laboratory, Middle East Technical University, Ankara, 0631 Turkey

**Keywords:** Zeolite, Enzyme, Biosensor, Creatinine deiminase, ISFET, BEA, Silicalite, Gold nanoparticle

## Abstract

The combination of advantages of using zeolites and gold nanoparticles were aimed to be used for the first time to improve the characteristic properties of ion selective field-effect transistor (ISFET)-based creatinine biosensors. The biosensors with covalently cross-linked creatinine deiminase using glutaraldehyde (GA) were used as a control group, and the effect of different types of zeolites on biosensor responses was investigated in detail by using silicalite, zeolite beta (BEA), nano-sized zeolite beta (Nano BEA) and zeolite BEA including gold nanoparticle (BEA-Gold). The presence of gold nanoparticles was investigated by ICP, STEM-EDX and XPS analysis. The chosen zeolite types allowed investigating the effect of aluminium in the zeolite framework, particle size and the presence of gold nanoparticles in the zeolitic framework.

After the synthesis of different types of zeolites in powder form, bare biosensor surfaces were modified by drop-coating of zeolites and creatinine deiminase (CD) was adsorbed on this layer. The sensitivities of the obtained biosensors to 1 mM creatinine decreased in the order of BEA-Gold > BEA > Nano BEA > Silicalite > GA. The highest sensitivity belongs to BEA-Gold, having threefold increase compared to GA, which can be attributed to the presence of gold nanoparticle causing favourable microenvironment for CD to avoid denaturation as well as increased surface area. BEA zeolites, having aluminium in their framework, regardless of particle size, gave higher responses than silicalite, which has no aluminium in its structure. These results suggest that ISFET biosensor responses to creatinine can be tailored and enhanced upon carefully controlled alteration of zeolite parameters used to modify electrode surfaces.

## Background

Creatinine is one of the most important analytes used for determination of kidney and muscular dysfunction and control of patients receiving hemodialysis. Its concentration in serum can rise from 35–140 μM to 1 mM during nephron malfunction. However, it can fall below 40 μM due to decreasing of muscle mass [1, 2]. It is usually determined by a spectrophotometric method based on the Jaffe’s reaction despite the high volume of sample and the poor selectivity of this method. HPLC and capillary electrophoresis have been also used for clinical determination of creatinine [3, 4]. These methods require expensive instruments, time-consuming sample pre-treatment and skilled persons to operate them. In addition, sensors and biosensors for creatinine determination have been proposed to reduce cost, time and complexity of routine analysis of biological fluids by being fast, reliable and portable methods allowing home testing. Creatinine biosensors developed are mostly based on either amperometric or potentiometric detection methods [5, 6]. The sensing performance of these biosensors is greatly affected by the immobilization of the bioselective element onto the transducer surface. The conventional methods of enzyme immobilization are physical adsorption, covalent binding, cross-linking and enzyme entrapment with polymers. These methods, however, may suffer from low reproducibility and poor spatially controlled deposition. Recently, inorganic materials such as clays, sol-gels and zeolites have been investigated to solve these problems.

Zeolites are inorganic solids with large surface areas and well-defined internal structures of uniform cages, cavities or channels of monodisperse dimensions [7]. In the field of biosensors, zeolites are promising materials for enzyme immobilization since they have a large surface area, thermal/mechanical stabilities, ion exchange capacity and controllable hydrophilicity/hydrophobicity. Recently, there have been an increasing number of researches on zeolite containing biosensors such as conductometric biosensors based on zeolite immobilized urease [8], natural clinoptilolite [9] and amperometric biosensor based on dealuminated zeolite [10]. Additionally, Phadare et al. presented that conjugation of gold nanoparticle and zeolite leads to enhanced biocatalytic activity compared to the free enzyme in solution. They believed that the interaction of gold nanoparticles and oxide zeolite particles have an inductive effect on the bioactivity of protein. This study also resulted in enhanced pH and temperature stability [11].

Gold nanoparticles have different properties from its bulk size such as biocompatibility, high specific surface area, high surface energy and high conductivity. They also offer numerous adsorption sites to enzymes, antibodies and proteins, which make them an ideal choice for biosensors [12]. Crumbliss et al. used colloidal gold as an immobilization matrix for the development of amperometric biosensor to detect glucose. They concluded that enzymes are tightly adsorbed onto gold nanoparticles and these nanoparticles provide a biocompatible surface that is suitable for immobilizing active enzymes onto electrodes. The same enzymes were shown to denature on bulk metal surfaces [13, 14]. Despite these advantages, there are only a few articles using gold nanoparticles in the field-effect transistor-based biosensors [15–19].

We have previously shown the development of new methods for potentiometric [20, 21], amperometric [22] and conductometric [8, 23] transducers based on immobilization of enzymes on zeolites. In our recent study, owing to the unique advantages such as easiness, fastness, inexpensiveness and ability to analyse the biological content as electrical signal of electrochemical biosensors with an additional advantage of miniaturized silicon-based semiconductor nature of field-effect transistor (FET)-based sensors, we have developed a novel approach for constructing an ion selective field-effect transistor (ISFET) device for creatinine monitoring using silicalite [24]. The objective of this study was to combine the advantages of using zeolites with different properties (i.e. structure and particle size) with an additional benefit of the ability to use zeolites as hosts for gold nanoparticles to be used for the first time to improve the characteristic properties of ISFET-based creatinine biosensors.

## Methods

### Materials

Tetrapropylammonium hydroxide (TPAOH; 25% in water), tetraethyl orthosilicate (TEOS; 98%) from Acros Organics, sodium aluminate (anhydrous, Fisher), sodium hydroxide (97%, J.T. Baker), tetraethylammonium hydroxide (TEAOH; Aldrich, 35% in water), Ludox HS-40 colloidal silica (SiO_2_, Sigma-Aldrich, 40% in water) and aluminium isopropoxide (98%, Aldrich) were used for zeolite synthesis. For the ion exchange procedure, gold(III)chloride (Aldrich) and sodium borohydride (Sigma-Aldrich) were used.

For biosensor studies, enzyme creatinine deiminase (EC 3.5.4.21) with the activity of 36 U/mg, bovine serum albumin (BSA; fraction V), glycerol 25% aqueous solution of glutaraldehyde (GA) and creatinine were purchased from Sigma-Aldrich. Diethylaminoethyl dextran (DEAE-Dextran) was from Fluka Biochemica and lactitol was from Fluka. The working buffer solution (KH_2_PO_4_-NaOH), pH 7.4, was prepared from reagents of Helicon.

### Design of Potentiometric Transducer and Measuring System

**Fig. 1:**
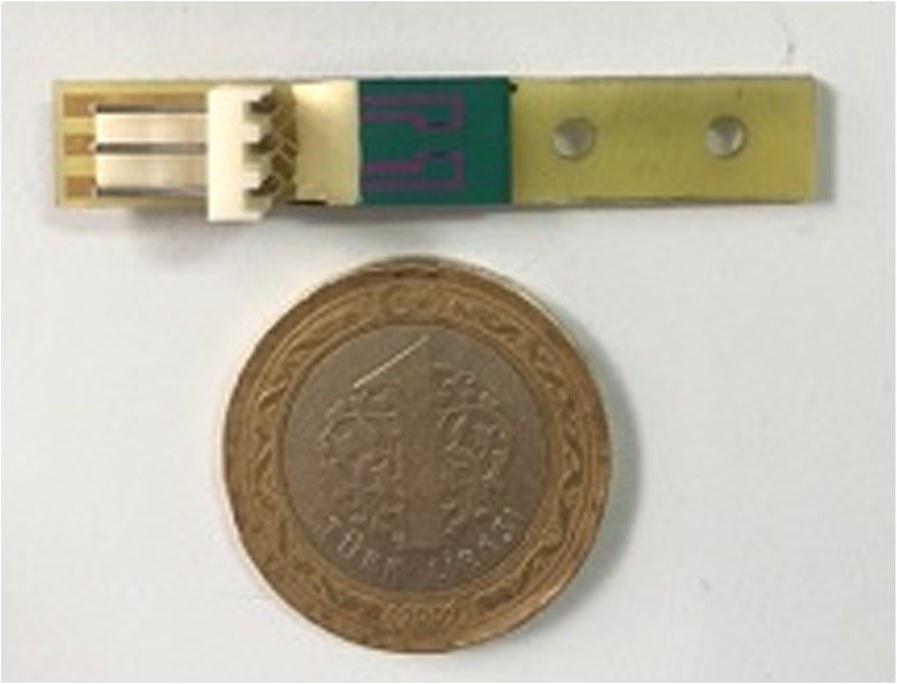
pH-sensitive field-effect transistor

pH-sensitive field-effect transistors used in the current study (Fig. 1) were fabricated at Lashkarev Institute of Semiconductor Physics of National Academy of Sciences of Ukraine (Kyiv, Ukraine) and the detailed information can be found in [25] and [26] respectively. Basically, the potentiometric biosensor presented has a differential pair of two identical p-channel field-effect transistors placed on a single crystal with the total area of 8 × 8 mm^2^. Usage of two transistors allows working in differential mode to avoid the changes associated with the fluctuations in temperature, environmental pH and electrical noise. The gate of the dielectric layer was formed from SiO_2_ and Si_3_N_4_ films. The transconductance of the ISFETs measured in phosphate buffer with Ag/AgCl reference electrode was 400–500 μA/V and pH sensitivity of the transistor was approximately 40 mV/pH, thus providing pH sensitivity of the transistor channel current of 15–20 μA/pH.

### Instruments

The synthesized zeolites were characterized by powder X-ray diffraction (XRD) using Rigaku-Ultima IV. Scanning electron microscopy (SEM) analyses were performed in a 400 Quanta FEI. Particle sizes of silicalite and nano BEA were estimated from SEM images whereas for zeolite BEA, Malvern Mastersizer 2000 was used. Cross-sectional SEM images were taken from zeolite-coated silicon wafers. High-angle annular dark-field (HAADF) STEM images of gold-BEA zeolite was obtained using a JEM JEOL 2100F electron microscope equipped with a field emission gun and operated at 200 kV with a STEM detector. This observation was coupled with EDX investigations for elemental analysis using Oxford Energy Dispersive Spectroscopy. The nitrogen adsorption/desorption experiments were carried out at by a Autosorb 6 series (Quantachrome Instruments) instrument. The surface area of the samples was obtained by Multipoint BET, while external surface area was obtained by a t-plot method. Sample preparation method includes outgassing samples under vacuum at 300 °C for 4 h before analysis. The elemental analysis of ion-exchanged and reduced samples was determined using Perkin Elmer Optima 4300DV ICP-OES. X-ray photo-electron spectroscopy (XPS) analysis was carried out on a PHI 5000 VersaProbe spectrometer with an Al-Kα radiation source. The binding energies were referenced to the internal standard C 1s binding energy at 284.5 eV. Contact angles, Ɵ, were measured from electrode surfaces using static sessile water drop method with an Attension Theta goniometer. For each sample, at least five measurements were made and the measurements were taken immediately after the drop had been deposited on the surface. The average angle was calculated by using the OneAttension software from both the left and right sides of the droplet. The standard error in Ɵ was approximately ±2°. Atomic force microscopy was performed in air on a Veeco MultiMode V AFM operated in tapping mode. A silicon tip was used with a scan rate of 1–2 Hz. For AFM measurements, zeolite-coated silicon wafers were used.

### Preparation of Biosensors for Creatinine Detection

#### Synthesis of Silicalite

The optimized molar composition of the gel used for synthesis of silicalite-1 is 1 TPAOH : 4 TEOS : 350 H_2_O. The alkali source used was TPAOH and the silica source was TEOS. TEOS was added to TPAOH solution under vigorous stirring. The mixtures were aged at room temperature for 6 h under stirring. The gel was introduced into Teflon-lined autoclaves for crystallization. Static synthesis was carried out for 1 day at 125 °C. Product was centrifuged three times at 7500 rpm and dried overnight at 50 °C. The particle size of silicalite is approximately 450 nm.

#### Synthesis of BEA

Optimized molar composition of the gel used for the synthesis of zeolite beta (BEA) is 1.92 Na_2_O : 1 Al_2_O_3_ : 60 SiO_2_ : 444 H_2_O : 4.6 (TEA)_2_O. The mixture of sodium aluminate, sodium hydroxide and distilled water was stirred for 40 min then placed in an oven at 100 °C for 50 min to form alumina precursor solution. TEAOH as a structure directing agent was added to the cooled mixture and stirred for 15 min. Finally, Ludox HS-40 colloidal silica as a source of silica were added into the prepared precursor solution and mixed for 15 min. The resulting mixture was introduced into Teflon-lined autoclaves for crystallization. Static synthesis was carried out for 7 days at 120 °C. Product was centrifuged three times at 7500 rpm and dried overnight at 50 °C. The average particle size of zeolite beta is 1–1.5 μm.

#### Synthesis of Nano BEA

Molar composition of the nano beta is 0.25 Al_2_O_3_ : 25 SiO_2_ : 490 H_2_O : 9 TEAOH. Silica source was TEOS. Aluminium isopropoxide, TEAOH and distilled water were used as the other reactants. Aging was continued under static conditions for 4 h with a clear solution. Crystallization was completed within 14 days under static conditions at 100 °C in Teflon-lined autoclaves. Product was purified by using centrifugation three times at 7500 rpm [27]. The particle size of nano beta is approximately 100 nm.

All zeolites used were calcined at 550 °C for 6 h at a rate of 1 °C/min to remove the templates and clear the pores.

#### Modification of BEA to BEA-Gold by Ion Exchange and Reduction

The ion exchange and reduction of zeolite beta were done by slight modification of the procedure found in the literature [28]. The synthesized zeolite beta was calcined at 500 °C in air for 6 h before ion exchange procedure. Four hundred milligrams of calcined BEA was added to 2.33 mM Au(III)chloride solution for obtaining gold ion-exchanged BEA samples (Au(III)BEA)) according to maximum theoretical loading of 4 wt.% Au at 50 °C with stirring. After 24 h, obtained Au(III)BEA samples were washed and centrifuged for four times at 7500 rpm. Following this, the samples were dried at 50 °C under ambient air. Au(0)BEA nanoclusters were produced from gold ion reduction at 50 °C in sodium borohydride (3.4 mM) suspension and called as BEA-Gold. The reduction step was terminated when the hydrogen gas formation was finished. BEA-Gold samples were washed and centrifuged for four times at 7500 rpm and dried at 50 °C. The gold content was measured as 0.65 wt.% using ICP-OES.

#### Immobilization of Enzymes in Glutaraldehyde Vapour

Working solution was prepared using 10% CD and 10% BSA in 20 mM phosphate buffer (pH 7.4) containing 10% glycerol, 4% lactitol and 0.4% DEAE-Dextran. In reference solution, instead of enzyme, 20% BSA was used to hold the protein concentration constant. BSA was used both for providing amino groups for cross-linking and stabilization of enzymes. Lactitol and DEAE-Dextran were used as enzyme stabilizers. The latter and glycerol were also used to prevent the membrane from being cracked with a view to providing better adhesion to ISFET surface.

0.1 μl of working solution was deposited to one gate area of the transducer, and 0.1 μl of reference solution was deposited on the other gate area of the transducer. These transducers were exposed to saturated GA vapour for 15 min and dried at ambient air for 15 min. Finally, they were washed with buffer solution to remove the unbound protein and excess GA.

#### Immobilization of Enzymes onto Zeolite Layers

**Fig. 2:**
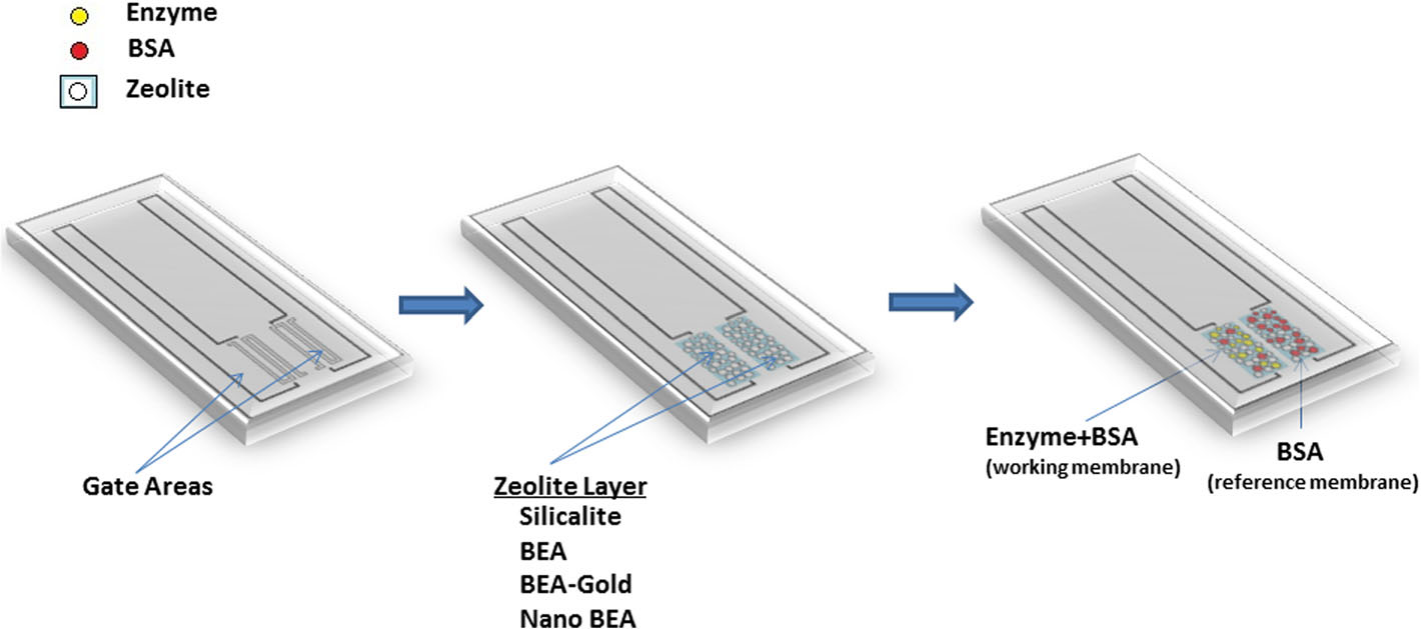
Creation of zeolite electrodes

The gate areas of the transducers were coated with different types of zeolites or BEA-Gold, and enzyme immobilization was performed by using reported adsorption method in [8]. The overall procedure is summarized in Fig. 2.

##### Drop-Coating of Zeolites onto Transducers

Different zeolite solutions with 10% concentration were prepared using 5 mM phosphate buffer (pH 7.4) and these solutions were ultrasonicated before usage to obtain a homogeneous mixture. 0.2 μl of zeolite mixture was deposited on each active zone of transducers and they were heated at 120 °C for 15 min. The obtained surfaces were washed with buffer solution to remove unbound zeolites before enzyme immobilization.

##### Adsorption of Enzymes onto Zeolite Layers

The working solution used for the adsorption method contained 10% CD solution in 20 mM phosphate buffer, pH 7.4 with 10% glycerol, 4% lactitol and 0.4% DEAE-Dextran whereas the reference solution contained 10% BSA instead of CD. 0.16 μl of working solution was deposited onto one gate area of the transducer, and 0.16 μl of reference solution was deposited onto the other gate area of the transducer. These transducers were dried in ambient air for 15 min.

#### Biosensor Measurements

All measurements were performed in an open stirred measuring cell filled with 5 mM phosphate buffer at pH 7.4. The substrate concentrations were varied by addition of a different volume of 500 mM stock solution to the working buffer. Each experiment was performed at least three times. Fluctuations of temperature, pH and voltage effects producing nonspecific changes in the output signal were avoided by usage of differential mode of measurements.

## Results and Discussion

### Characterization of Synthesized Zeolites

The basic characteristics of four types of zeolites such as structure, Si/Al ratio, particle size, surface area, external surface area and zeta potential values are presented in Table 1.

**Table 1:** Physicochemical characteristics of used zeolites

Sample name	Structure	Si/Al^a^	Particle size^b^	*S*_BET_, (m^2^/g)^c^	*S*_ext_, (m^2^/g)^d^	Zeta potential, (mV)^e^
Silicalite	MFI	No Al.	~470 nm	447	96	−62.6
BEA	BEA	21.5 ± 0.7	~1.2 μm	743	128	−47.9
BEA-Gold	BEA	21.0 ± 0.9	~1.2 μm	776	160	−47.2
Nano BEA	BEA	20.8 ± 0.8	~100 nm	696	183	−36.6

^a^Measured by EDX

^b^Measured by SEM and Master sizer

^c^Measured by Multipoint BET

^d^Measured by t-plot method

^e^Measured by zeta potential at pH 7

According to Table 1, silicalite which belongs to MFI-type structure has the lowest surface area (447 m^2^/g) with the lowest zeta potential (−62.6 mV) and it has no aluminium inside whereas BEA-type of zeolites have similar Si/Al ratios with different particle sizes such as 1.2 μm for BEA and 100 nm for nano BEA. BEA-Gold has the highest surface area (776 m^2^/g) which is a result of the increase almost only in external surface area of BEA (an increase of about 20%). Additionally, nano BEA has the lowest zeta potential as −36.6 mV.

**Fig. 3:**
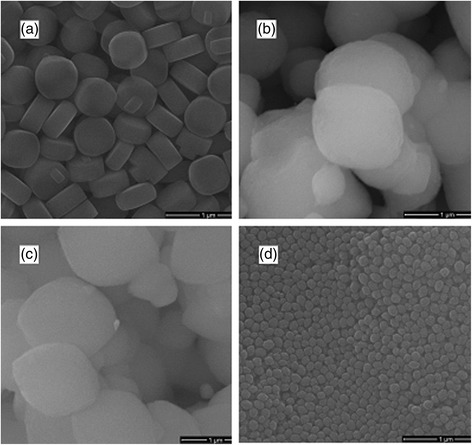
The SEM images of **a** silicalite, **b** BEA, **c** BEA-Gold and **d** nano BEA

Representative SEM images are shown in Fig. 3 for (a) silicalite, (b) BEA, (c) BEA-Gold and (d) Nano BEA. The crystal morphology for silicalite was shown to be more of a round plate shape of around 400–450-nm length and 250-nm thickness with clear surface. Nano BEA crystals were around 100 nm with more of a spherical morphology (Fig. 3b). BEA samples were around 1 μm with typical truncated bipyramidal morphology. It can be seen that this morphology for zeolite BEA remained the same after reduction procedure in BEA-Gold.

**Fig. 4:**
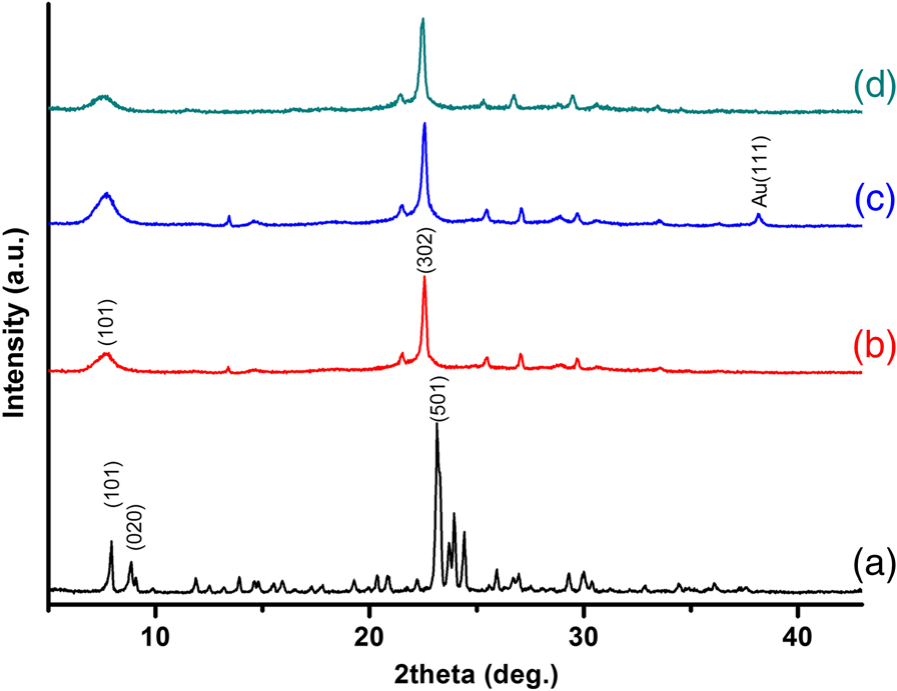
The X-ray diffraction patterns of *a* silicalite, *b* BEA and *c* BEA-Gold and *d* nano BEA

X-ray diffraction patterns of silicalite, BEA, BEA-Gold and nano BEA are presented in Fig. 4 (a–d). It was seen that all the peak positions were matching with the literature. In Fig. 4 (a), the characteristic diffraction peaks of silicalite at 7.8, 8.8 and 23.1° 2*θ*, which are similar to reference [29], correspond to (101), (020) and (501) reflections, respectively. Two characteristic diffraction peaks of zeolite BEA at Bragg angles of 7.6 and 22.6° 2*θ* which correspond to (101) and (302) reflections, respectively, were seen in X-ray diffraction patterns of BEA, BEA-Gold and nano BEA as presented in Fig. 4 (b–d) [30, 31]. The results of the XRD were in accordance with the SEM images, which show successful synthesis of silicalite, BEA and nano BEA. Additionally, X-ray diffraction pattern of BEA-Gold given in Fig. 4 (c) indicates that there is no noticeable change in the positions of the Bragg peaks demonstrating that the BEA-Gold structure was fully retained with a slight feature of Au^0^ phase at 38.1° 2*θ* [32].

Additional insight for gold characterization in zeolite BEA was investigated by ICP-OES, STEM, STEM-EDX and XPS. According to ICP-OES, BEA-Gold contains 0.65% gold, whereas zeolite BEA has no gold inside. In Table 2, it can be noticed that Si/Al ratio of the zeolite remains approximately the same after ion exchange and reduction procedure, which suggests that the local structure of zeolite BEA did not collapse.

**Table 2:** ICP results of BEA and BEA-Gold

ICP	BEA	BEA-Gold
Si(%)	35.80 ± 1.6	34.70 ± 0.7
Al(%)	1.52 ± 0.03	1.52 ± 0.02
Na(%)	0.45 ± 0.01	0.03 ± 0.01
Au(%)	–	0.65 ± 0.02
Si/Al (mole)	23.55	22.83

**Fig. 5:**
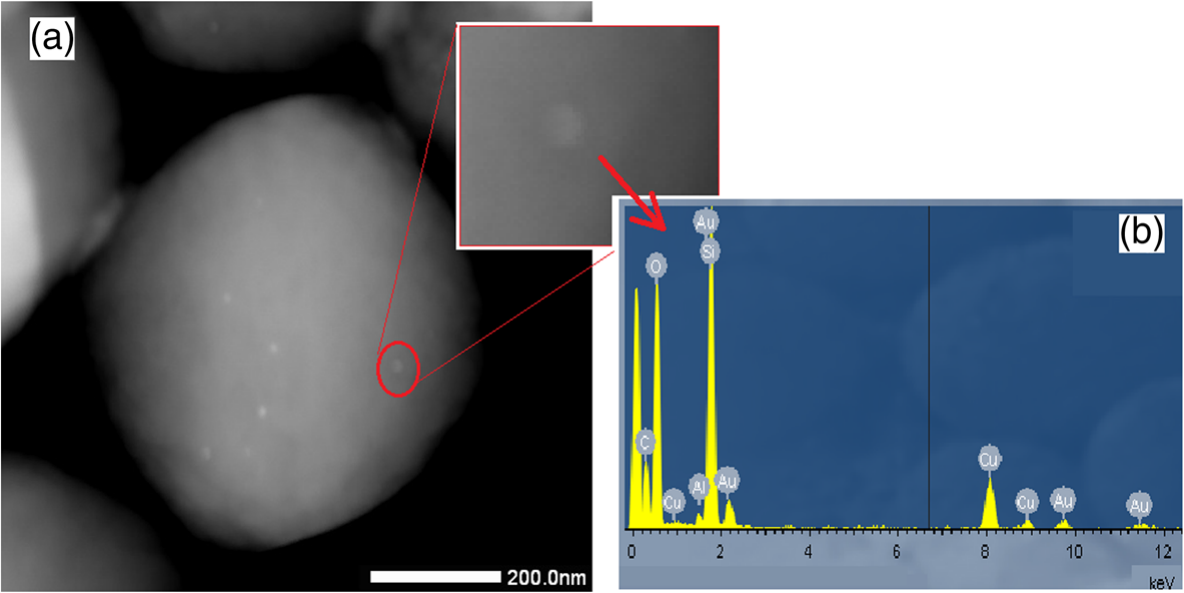
**a** STEM dark-field image of gold nanoparticles and **b** STEM-EDX spectra of gold nanoparticle selected in **a**

The presence of gold nanoparticles can be seen in STEM images presented in Fig. 5. The heavy Au atoms clearly stand out on the light background of zeolite BEA. Additionally, STEM-EDX at the shown point of Fig. 5a can be shown as additional evidence for the existing gold nanoparticles. The average size of gold nanoparticles produced was 10 nm according to the taken STEM images. This result showed that gold nanoparticles were on the zeolite surface rather than in the pores of zeolite since the pore diameters are 5.6 × 5.6 Å and 7.7 × 6.6 Å for zeolite BEA.

**Fig. 6:**
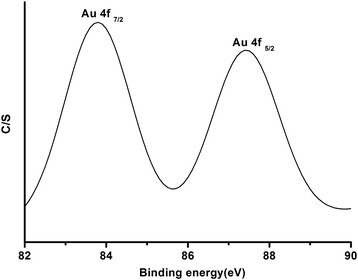
XPS spectrum in the region 82–90 eV of BEA-Gold (0.65 wt.% Au)

The XPS spectrum of BEA-Gold given in Fig. 6 shows two bands at 83.8 and 87.4 eV, corresponding to Au(0)4f_7/2_ and Au4f_5/2_, respectively. The band positions show good correspondence with literature [28, 33].

Cross-sectional SEM images of (a) silicalite, (b) BEA, (c) BEA-Gold and (d) nano BEA on silicon wafers are presented in Table 3 with corresponding AFM images and surface roughness values. As seen from the cross-sectional SEM images, the thickness of the coated zeolite layer was in the range of 10–20 μm. According to the obtained AFM images, electrode surfaces modified with nano BEA has the smoothest surface with a surface roughness value of 0.08 ± 0.01 nm. On the other hand, BEA-Gold, BEA and silicalite have similar surface roughness values of 48.85 ± 8.3, 42.00 ± 4.9, 44.45 ± 4.03, respectively.

**Table 3:**
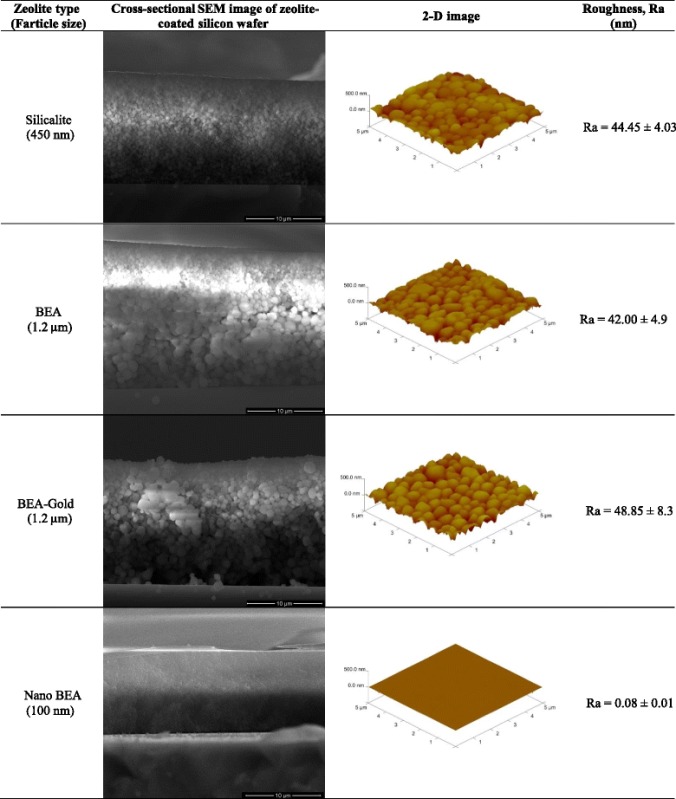
Cross-sectional SEM and AFM images of silicalite, BEA, BEA-Gold and nano BEA on silicon wafer with their representative surface roughness values (Ra)

According to the contact angle measurements presented in Table 4, zeolite-coated surfaces were becoming more hydrophilic when compared to GA cross-linked surfaces. The contact angles were in the following order: BEA = BEA-Gold < Nano BEA < Silicalite < GA. This is an expected result, since it was known that a decrease in Al^+3^ content, which is related to ion exchange capacity of a zeolite, produces more hydrophobic nature [34].

**Table 4:** Contact angles of biosensor surfaces

Sample name	Contact angle, *Ɵ* (°)
Plain surface	56
GA	66
Silicalite	29
BEA	6
BEA-Gold	5
Nano BEA	15

### Characteristics of Zeolite-Modified ISFET Biosensors

The creatinine-sensitive biosensor based on ion sensitive field-effect transistor (ISFET) operates according to the hydrolysis reaction of creatinine catalysed by creatinine deiminase:

$$ \begin{array}{l}\kern3.62em \mathrm{Creatinine}\;\mathrm{deiminase}\\ {}\mathrm{Creatinine} + {\mathrm{H}}_2\mathrm{O}\ \to\ {{\mathrm{N}\hbox{-} \mathrm{methylhydantoine} + \mathrm{NH}}_4}^{+}\end{array} $$

The enzymatic reaction occurring at the dielectric gate of the transducer produces a pH increase that is proportional to creatinine concentration, which makes it detectable by the currently developed ISFET device [2].

**Fig. 7:**
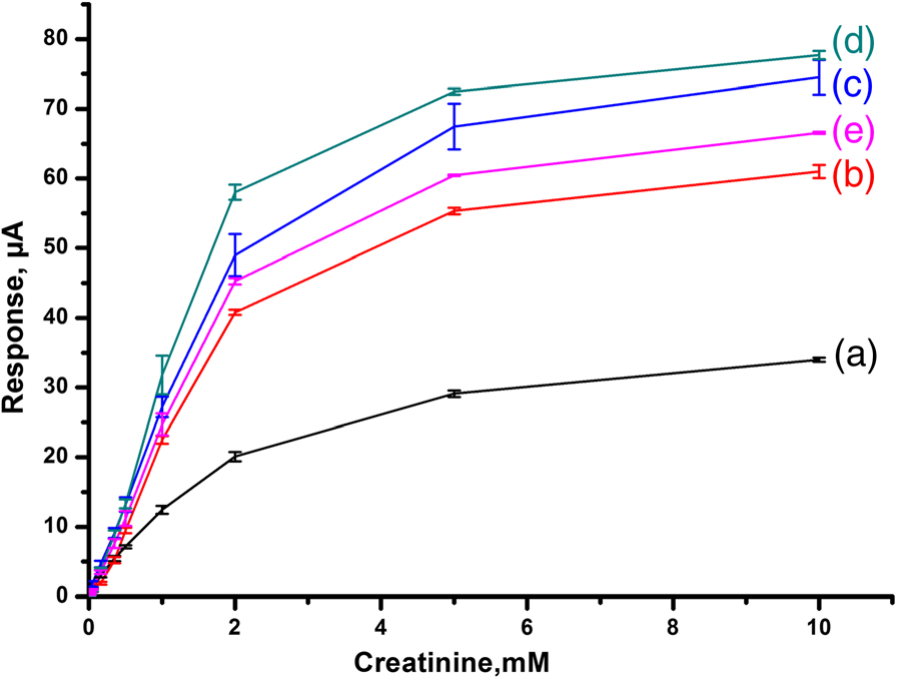
The calibration curves for determination of creatinine obtained with biosensors created by CD immobilization; *a* in GA vapour, adsorption on: *b* silicalite, *c* BEA, *d* BEA-Gold and *e* Nano BEA. The measurements were carried out in 5 mM phosphate buffer solution (pH 7.4)

In this study, GA cross-linking, the traditional method of enzyme immobilization technique, was used as a control group to compare the effect of zeolites on characteristics of zeolite-modified potentiometric biosensors such as sensitivity, linear range, response time, detection limit and regeneration time. Therefore, firstly, the calibration curves for all biosensors up to 10 mM of creatinine concentration were obtained and presented in Fig. 7. As seen in Fig. 7, all zeolite-based biosensors regardless of type had higher sensitivities than GA-based biosensor. The sensitivities of the biosensors decreased in the order of BEA-Gold > BEA > nano BEA > Silicalite > GA. BEA zeolites, having aluminium inside, regardless of its particle size, gave higher response than silicalite which has no aluminium in its structure. This result is consistent with our previous results showing that the sensitivity of urease biosensor based on ISFET developed by nano BEA coating is higher compared to silicalite-coated one [20] and the same trend was observed for amperometric glucose biosensor [22]. This result can be attributed to the presence of aluminium in zeolite BEA and nano BEA, providing both hydrophilicity and Brønsted acid sites (Si–OH–Al) which are strongly effective in protein adsorption [35, 36]. Additionally, Tavolaro et al. studied the effect of different acidity owing to zeolite framework on protein immobilization and concluded that the protein immobilization is proportional to effective acidity (*A*_E_) which is defined as:

$$ {A}_{\mathrm{E}} = m\ {\alpha}_0 $$

where *m* = Al/Al + Si and *α*_0_ is the efficiency coefficient [36]. In their study, they found the immobilization trend as FAU > BEA > MFI, which is similar to what was observed in the current study (BEA > MFI).

Biosensor responses were compared for BEA and nano BEA samples to investigate the effect of particle size, since both zeolites had similar Si/Al ratios and identical zeolite framework (BEA). It was observed that the BEA-based one having a higher surface area (743 m^2^/g) with larger particle size (~1.2 μm) showed higher response. This result can also be related with its surface roughness since enzymes prefer rough surfaces rather than smooth surfaces [37–39].

In the current study, more hydrophilic surfaces gave higher sensitivities as consistent with the findings of Peng et al. They demonstrated that enzyme adsorption can be more on hydrophobic surfaces whereas hydrophilic surfaces can be more active enzymatically [40].

Linear dependence between response current and creatinine concentration, the sensitivities of the obtained biosensors with the other characteristics such as detection limit (at an S/N of 3), response time and regeneration time for cross-linked and zeolite-coated-based biosensors are presented in Table 5. As it can be seen, all zeolite-based biosensors silicalite, BEA, BEA-Gold and nano BEA showed good linear dependence with correlation coefficients of 0.9934, 0.9976, 0.9967 and 0.9980, respectively. The highest sensitivity belongs to BEA-Gold, having a threefold increase compared to GA, this can be attributed to the gold presence causing favourable microenvironment for CD to avoid denaturation as well as increased surface area for interaction. Furthermore, when comparing BEA and BEA-Gold-based biosensors, it was observed that an increase in external surface area of about 20% resulted in approximately similar increase in the sensitivity.

**Table 5:** Characteristics of creatinine-based biosensors with different types of enzyme immobilization

Material	Linear equation between response (*y*) and creatinine concentration (*x*)	Sensitivity, μA/mM	Detection limit, μM	Response time, s	Regeneration time, s
GA	*y* = 10.131*x* + 0.9594, *R* ^2^ = 0.9807	10	10	240	300
Silicalite	*y* = 20.79*x* − 0.3002, *R* ^2^ = 0.9934	21	5	90	80
BEA	*y* = 24.663*x* + 0.6437, *R* ^2^ = 0.9976	25	5	150	180
BEA-Gold	*y* = 29.591*x* − 0.3703, *R* ^2^ = 0.9967	30	5	150	120
Nano BEA	*y* = 22.942*x* + 0.0394, *R* ^2^ = 0.9980	23	5	120	120

The linear dynamic range (up to 2 mM of creatinine) was same for all biosensors; however, there was a significant improvement in response time of about 60% decrease in silicalite-based biosensors and a minimal 40% decrease in regeneration time of all BEA-type of zeolite-based biosensors compared to respective values of GA-based biosensors. Additionally, the detection limit was increased twofold for all types of zeolite-based biosensors compared to GA-based biosensors.

**Fig. 8:**
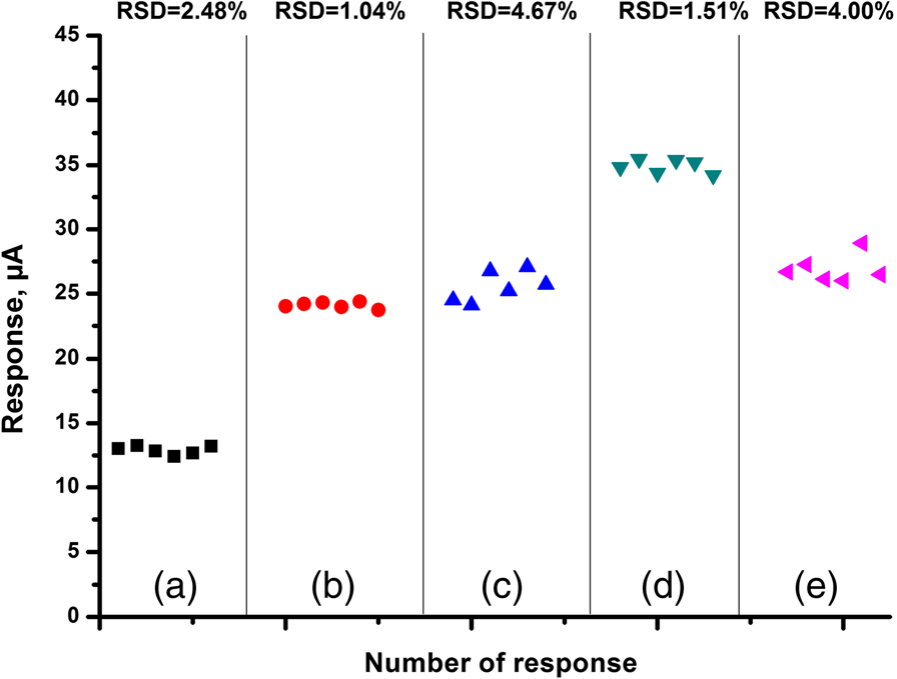
Reproducibility of signals obtained with biosensors created by CD immobilization; *a* in GA vapour, adsorption on: *b* silicalite, *c* BEA, *d* BEA-Gold and *e* Nano BEA. The measurements were carried out in 5 mM phosphate buffer solution (pH 7.4)

The stability of the created biosensors was investigated through reproducibility and inter-reproducibility studies. In reproducibility studies, the responses of the biosensors to 1 mM concentration of creatinine were measured over one working day with 30–35-min time intervals and continuous stirring at room temperature to determine signal repeatability. During this study, after obtaining the response, the working cell was rinsed with buffer three times to discard the products of enzymatic reaction on the biosensor surface. This measurement was repeated for six times for every type of immobilization. Finally, the relative standard deviations (RSD) for determining creatinine are presented in Fig. 8. As seen, the tested biosensors had rather good reproducibility as a sign of stable operation. The RSD values for all biosensors were in the range 1–5% with BEA having the highest RSD value of 4.67%, while the lowest RSD value belongs to the silicalite-based biosensors (1.04%).

For inter-reproducibility studies, several clean electrodes were drop-coated with zeolites then CD was adsorbed on these transducers as described in the chapter titled ‘Immobilization of enzymes onto zeolite layers’. After obtaining the response to 1 mM of creatinine in three repetitions, the enzyme and zeolites were removed from the transducers with ethanol-wetted cotton. Thereafter, the whole procedure was repeated three times and RSD values of biosensors were calculated. According to the results, the highest RSD (36.03%) belonged to the traditional method prepared with GA and the least RSD belonged to BEA-Gold (3.19%), while the intermediate values belonged to Silicalite (13.27%), BEA (10.7%) and Nano BEA (18.5%). The low RSD for zeolite-based biosensors compared to GA-based biosensors can be due to the better controlled procedure of adsorption. Additionally, low RSD of BEA-Gold can be due to the presence of gold nanoparticles, which will bind covalently the amine groups of the enzyme and actively stabilize the enzyme. This result is consistent with the study of Gole et al., where pepsin-gold colloid conjugates were prepared by just mixing under protein-friendly conditions and then demonstrated that pepsin showed significant catalytic activity. They explained the binding mechanism of the enzyme to the gold nanoparticle by covalent interactions between thiol groups in cysteine residues as well as amine groups in lysine residues of pepsin with the surface of gold. This interaction resulted in a more stabilized enzyme with its activity [41].

## Conclusions

In this study, creatinine-based ISFET biosensors have been successfully produced by modifying the gate of ISFET with four different types of zeolites separately. Their responses and other biosensor characteristics such as sensitivity, detection limit, response time and regeneration time are compared with covalently cross-linked CD as a control group. All zeolite-based biosensors had higher sensitivities, lower detection limits, response and regeneration time than GA-based biosensors with good reproducibility showing that zeolites can be good alternatives for creatinine biosensor production. The incorporation of zeolite and gold nanoparticles for adsorption of CD on ISFET-based biosensor was studied for the first time. The sensitivities of the obtained biosensors were decreasing in following order: BEA-Gold > BEA > nano BEA > Silicalite > GA. The BEA-Gold-based biosensor resulted in threefold increased sensitivity compared to GA-based biosensor, which could be attributed to favourable microenvironment for CD to avoid denaturation as well as increased surface area produced by gold nanoparticles with good stability. This result showed that gold nanoparticles can be used with zeolite to improve the characteristics of ISFET-based biosensors.
